# Immune checkpoints and immunotherapy for colorectal cancer

**DOI:** 10.1093/gastro/gov053

**Published:** 2015-10-27

**Authors:** Preet Paul Singh, Piyush K. Sharma, Gayathri Krishnan, A. Craig Lockhart

**Affiliations:** ^1^Division of Oncology, Washington University School of Medicine, St. Louis, MO, USA and; ^2^Department of Surgery, Washington University School of Medicine, St. Louis, MO, USA

**Keywords:** immunotherapy, colorectal cancer, checkpoint inhibition/blockade, programmed death 1 (PD-1), cytotoxic T-lymphocyte-associated antigen 4 (CTLA-4), vaccine, pembrolizumab

## Abstract

Colorectal cancer (CRC) remains one of the major causes of death worldwide, despite steady improvement in early detection and overall survival over the past decade. Current treatment paradigms, with chemotherapy and biologics, appear to have reached their maximum benefit. Immunotherapy, especially with checkpoint inhibitors, has shown considerable clinical benefit in various cancers, including mismatch-repair-deficient CRC. This has led to the planning and initiation of several clinical trials evaluating novel immunotherapy agents—as single agents, combinations and in conjunction with chemotherapy—in patients with CRC. This article reviews biological and preclinical data for checkpoint inhibitors and discusses various immunotherapy trials in CRC, as well as current efforts in CRC immunotherapy.

## Introduction

Colorectal cancer (CRC) is the third most common cancer in males and the second most common in females [1]. At the time of diagnosis, more than 20% of patients have metastatic disease and the survival rate of CRC patients with distant metastasis is estimated to be 12% [2–4]. Although, due to significant strides in surgical management, chemotherapy and biological therapy, average survival for advanced disease now approaches 30 months, metastatic CRC still remains the fourth most common cause of death from cancer.

The significance of the immune system in the biology of CRC is emphasized by retrospective assessments of immune infiltrates in resected CRC tumors. The presence of tumor infiltrating lymphocytes—especially CD8 + lymphocytes—in the tumor microenvironment, as well as regional lymph nodes, has been linked to better prognosis [5]. Also, increased infiltration of specific regions of the CRC tumors by cytotoxic memory T lymphocytes (CD8 + CD45RO + T-cells) is highly correlated with reduced risk of recurrence of CRC and better survival [6, 7]. It is also known that the presence of these effector memory T-cells is more important than naïve T-cells in reducing the risk of relapse and improving survival. The prognostic significance of T-cells—unlike other inflammatory cells—argues that cancer immunotherapies modulating T-cell responses could lead to improved survival.

Recent developments in immune-biotechnology and the discovery of immunotherapy agents—specifically checkpoint inhibitors—have been promising for treatment of melanoma [8, 9] and non–small cell lung cancer [10], leading to regulatory approval of these novel drugs. The significant activity of these agents in various epithelial tumors raises the prospect that the immune system may represent a favorable avenue for advancing the management of patients with CRC. This review focuses on current utilization of checkpoint blockade, as well as other immunotherapy agents, and their potential integration into existing therapeutic regimens for advanced colorectal cancers.

## Immune checkpoints: biology and preclinical studies

**Figure 1.**
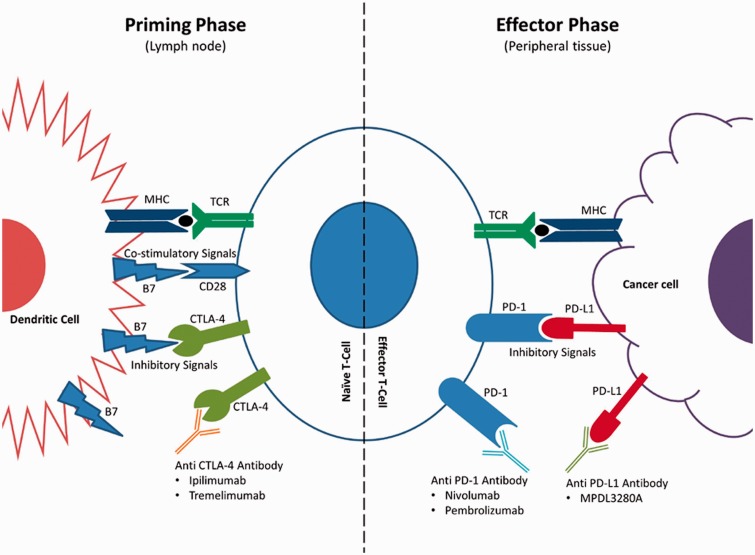
Checkpoint blockade in tumor immunotherapy. T-cells initiate the immune response though recognition of antigenic peptides presented by the MHC on the surface of antigen-presenting cells and cancer cells through their TCR. TCR engagement alone is insufficient to turn on a T-cell response, and additional co-stimulatory signal via B7 are required for cytokine production, targeT-cell lysis and effector cell responses. B7 protein can pair with CD28 on T-cells, leading to amplification of TCR signaling, or CTLA-4 on a T-cell inhibiting T-cell activation. PD-1 primarily inhibits effector T-cell activity in the effector phase within tissue and tumors—unlike CTLA-4, which mainly modulates early steps in T-cell activation. PD-1 inhibitory receptor is expressed by T-cells during long-term antigen exposure and results in inhibition of T-cells on interaction with PD-L1, which is expressed in the tumor microenvironment. Immune checkpoint blockade via monoclonal antibodies leads to preferential activation of cancer-specific T-cells and revival of tumor immunity (MHC: major histocompatibility complex; TCR: T-cell receptor; CTLA-4: cytotoxic T-lymphocyte-associated antigen 4; PD-1: programmed death 1; PD-L1: programmed death-ligand 1).

T-lymphocytes act as the chief effector cells in the immune response against tumor cells by recognizing and mounting a cytotoxic response against antigenic molecules arising out of the genetic and epigenetic alterations that mark malignant transformation. The immune response is initiated by the recognition of antigenic peptides presented by the major histocompatibility complex (MHC) on the surface of antigen-presenting cells by the T-cell receptor (TCR). TCR engagement alone is insufficient to lead to the clonal expansion and differentiation required for an effective and lasting immune response against the offending antigen. Additional co-stimulatory signals are required for cytokine production, targeT-cell lysis and effector cell responses (Figure 1). There also exists an intricate system of inhibitory signals (immune checkpoints), which is crucial in the maintenance of peripheral immune-tolerance and preventing auto-immunity. The amplitude and duration of the T-cell response depends on the balance between co-stimulatory and inhibitory signals. T-cell responses may be amplified by agonists of co-stimulatory receptors or antagonists of inhibitory signals. As the immune checkpoints are often co-opted by tumors to escape immune surveillance, inhibitors of immune checkpoints lead to revival of tumor immunity. The two checkpoint targets that have been studied more extensively in cancer are the cytotoxic T-lymphocyte-associated antigen 4 (CTLA-4) and the programmed death 1 (PD-1) receptor (Figure 1). Other targets, like lymphocyte activation gene 3 (LAG-3), are also being studied and early phase clinical trials are under way.

### Cytotoxic T-lymphocyte-associated antigen 4

There are several membrane protein ligands on antigen presenting cells (APC), which interact with specific receptors on T-cells to produce a co-stimulatory signal or a co-inhibitory signal to enhance or decrease the T-cell immune response. B7 protein found on activated APC can pair with CD28 on T-cells, leading to amplification of TCR signaling, or cytotoxic T-lymphocyte-associated antigen 4 (CTLA-4) (CD152) surface protein on a T-cell inhibiting T-cell activation and cytokine production with induction of a state of anergy and proliferative arrest [11].

The crucial role of CTLA4-B7 interaction in immune homeostasis is demonstrated by the fact that CTLA-4 knockout mice develop lethal lymphoproloferative disorders [12]. Regulatory T-lymphocytes (T_reg_) exhibit the constitutive expression of CTLA-4 and their suppressive function *in vivo* can be suppressed by blocking CTLA-4 [13, 14]. Allison *et al*.** reported in 1996 that blocking CTLA-4 function could not only enhance rejection of transplanted colon carcinoma in mice, but also delay the growth of established tumors [15]. In a CT-26 colon carcinoma model, a combination of an anti-CTLA-4 monoclonal antibody with ixabepilone or paclitaxel resulted in a 50–70% tumor rejection rate [16]. An enhanced anti-tumor response at the primary site—as well as an abscopal effect—was observed when fractionated radiotherapy was combined with an anti-CTLA-4 monoclonal antibody in a murine colon cancer model MCA38 [17]. In another study that utilized the same model, a combination of anti-CTLA-4 and anti-4-1BB-enhanced CD8 T-cell mediated anti-tumor response and significantly reduced liver metastasis when compared with treatment utilizing either antibody alone [18].

### Programmed death 1

Programmed death 1 (PD-1) primarily inhibits effector T-cell activity in the effector phase within tissue and tumors—unlike CTLA-4, which mainly modulates early steps in T-cell activation [19]. PD-1 binds to two distinct members of the B7 family: programmed-death ligands 1 and 2 (PD-L1 and PD-L2). PD-L1 has a very broad expression range, which includes hematopoietic cells such as dendritic cells (DC), macrophages, T-cells and B cells, as well as non-hematopoietic cells such as epithelial and endothelial cells [20, 21]. PD-L2 has a more restricted expression profile limited to macrophages, DC and masT-cells. PD-1-deficient mice develop a delayed-onset, organ-specific auto-immunity, which is in contrast with the rapid-onset systemic autoimmunity that characterizes CTLA-4-deficient mice [22]. When BALB/c mice bearing CT-26 colon tumors were treated with anti-PD-1 antibodies as single-agents, there was growth retardation but no eradication of tumors, which notably could be accomplished with dual blockade of PD-1 and CTLA-4 [23]. Iwai *et al*.** intravenously injected PD-1 knockout mice (PD-1^-/-)^ and wild-type (WT) mice with CT26 colon cancer cells to mimic metastatic spread, and found that tumor formation in the lungs was significantly reduced in the PD-1^-/-^ mice. Treatment with anti-PD-1 antibodies also had the same effect [24]. The addition of anti-PD-L1 antibodies was reported to potentiate the survival benefit imparted by IL-15 in a metastatic colorectal cancer murine model. The greatest survival benefit in this study was observed when IL-15 was combined with anti-PD-L1 and anti-CTLA-4 treatment [25]; in a syngeneic murine colon cancer model, anti-PD-L1, when combined with ionizing radiation, effectively controlled tumor growth, which could not be achieved with either treatment alone, indicating synergy or an abscopal effect with radiation therapy [26].

While single or dual checkpoint blockade causes significant improvements in anti-tumor immune response, there is potential to further boost this response with additional immune-sensitizing strategies. In one study, treatment with anti-PD-1 or anti-PD-L1 or anti-CTLA4 alone caused CT-26 colon tumors to be rejected in 25%, 33%, and 50% of the mice injected, respectively, which increased to 75% with dual blockade. Remarkably, a complete (100%) tumor rejection was observed when dual blockade was combined with a cancer vaccine, GVAX [27].

### Lymphocyte activation gene 3

Lymphocyte activation gene 3 (LAG-3) is another molecule expressed on activated T-cells, with diverse biological effects on T-cell function. Its main ligand is MHC class II, and LAG-3/MHC class II interaction down-regulates antigen-dependent stimulation of CD4+ T lymphocytes [28]. The protein negatively regulates the cellular proliferation, activation, and homeostasis of T-cells in a similar fashion to CTLA-4 and PD-1, and has been reported to play a role in the T_reg_ suppressive function [29–31]. LAG-3 also helps to maintain CD8+ T-cells in a tolerogenic state [32] and, working with PD-1, helps to maintain CD8 exhaustion during chronic viral infection [33].

## Immunotherapy for colorectal cancer

### Non-specific immunotherapy and immunomodulatory effects of chemotherapy

Cytokines such as interferon (IFN), interleukins and granulocyte macrophage colony-stimulating factor (GM-CSF) constitute non-specific immunotherapy, which augments host immunity against tumor antigens. Conventional chemotherapies also may have some effect through the immune system. Oxaliplatin triggers a form of cell death that is thought to be immunogenic, whereas the chemical analogue cisplatin does not trigger the same form of immunogenic cell death. In preclinical models, injection with oxaliplatin-killed CRC cells enhances the survival of mice that are subsequently challenged with live CRC cells and this protection requires an intact immune system [34]; thus the anti-tumor activity of oxaliplatin may also be related to its efficacy as an immune-modulatory agent and not solely as a cytotoxic drug.

A Phase II trial of gemcitabine, oxaliplatin and 5-fluorouracil (GOLF), combined with IL-2 and GM-CSF immune-adjuvant regimen (GOLFIG) in patients with CRC showed an overall response rate (ORR) of 56.5% and mean overall survival (OS) of nearly 19 months. Autoimmunity and substantial increase in lymphocyte count were found to be predictors for OS [35]. The GOLFIG-2 chemo-immunotherapy trial was a Phase II I multicenter study comparing GOLFIG with 5-fluorouracil/leucovorin plus oxaliplatin (FOLFOX) in chemotherapy-naïve, metastatic CRC patients. The study suffered early termination because of poor recruitment in the control arm, but GOLFIG showed superiority over FOLFOX in terms of progression free survival (PFS) and ORR, with a trend towards improvement of OS [36]. These findings provide proof-of-principle that chemo-immunotherapy may represent a novel option for first-line treatment of metastatic CRC.

## Immune checkpoint inhibitors

The development of checkpoint inhibitors is largely responsible for the recent excitement about immunotherapy. These agents are blocking antibodies to inhibitory cell-surface molecules, such as CTLA4 and PD-1/PD-L1, which restrain the priming and effector phases, respectively, of the adaptive T-cell immune response. The PD-1 pathway is a negative feedback system that represses Th1 cytotoxic immune responses. It is up-regulated in many tumors and, in their surrounding microenvironment and its blockade with antibodies to PD-1 or its ligands, has led to remarkable clinical responses in patients with various types of cancer [10, 37–39]. The expression of PD-1 ligands (PD-L1 or PD-L2) on the surface of tumor cells or immune cells is an important—but not a definitive—predictive biomarker of response to PD-1 blockade [38–41]. The clinical activity of checkpoint inhibitors in CRC needs to be evaluated in larger numbers of patients, in trials that examine predictive biomarkers, as well as combine these agents with conventional therapies. Table 1 summarizes the results of checkpoint inhibitor clinical trials in patients with CRC.

**Table 1.** Checkpoint inhibition in colorectal cancer: clinical trials

Author	Trial phase	Therapy	Patient population	Objective response rate	Overall survival	Progression-free survival	Other findings
Le *et al.* [43]	Phase II non-randomized, 3 centers	Pembrolizumab (anti PD-1) 10 mg/kg IV every 14 days	Metastatic CRC, pre-treated with ≥2 regimens Cohort A : MMR deficient CRC (11 patients) Cohort B : MMR proficient CRC (21 patients)	Cohort A: 40% (4/10 patients) Cohort B: 0% (0/18 patients)	Cohort A: Median OS not reached Cohort B: Median OS - 5 months	Cohort A: Median PFS not reached Cohort B: Median PFS – 2.2 months	High somatic mutation load associated with prolonged PFS (*P* = 0.02).
Topalian *et al.* [37]	Phase I	Nivolumab (anti PD-1) at doses of 1, 3, or 10 mg/kg every 2 weeks (in dose escalation phase)	Heavily pre-treated, metastatic CRC (*n* = 17 patients)	CR: 1 patient	-	-	The only responding patient had deficient MMR and PD-L1 positive tumor
Brahmer *et al.* [44]	Phase I	BMS-936559 (anti PD-L1) at doses of 0.3, 1, 3, and 10 mg/kg on days 1, 15, and 29 of 6-week cycle	Pre-treated, metastatic CRC (*n* = 18 patients)	No objective responses	-	-	-
Herbst *et al.* [40, 45]	Phase I	MPDL3280A (anti-PD-L1) at doses of≤1, 3, 10, 15 and 20 mg/kg IV every 3 weeks	Pretreated CRC (*n* = 4 patients)	PR: 1 patient	-	Ongoing response at 60 weeks	Patient post-treatment after 48 weeks
Bendell *et al.* [46]	Phase Ib	MPDL3280A (anti-PD-L1) Arm A: MPDL3280A 20 mg/kg + bevacizumab 15 mg/kg every 3 weeks Arm B: MPDL3280A 14 mg/kg + mFOLFOX6 + bevacizumab 10 mg/kg every 2 weeks	Arm A: Pretreated, metastatic CRC (≥3 prior regimens) (*n* = 14 patients) Arm B: Oxaliplatin naïve metastatic CRC (*n* = 30 patients; 23/30 as first line therapy)	Arm A: 8% (1/13) PR: 1 patient SD: 9 patients PD: 3 patients Arm B: 40% (12/30) PR: 12 patients SD: 14 patients PD: 4 patients	-	For responders, PFS range 10–61 weeks	Combinations well tolerated. No worsening of bevacizumab or chemotherapy-associated adverse events.
Chung *et al.* [55]	Phase 2, single-arm, multicenter	Tremelimumab (anti-CTLA-4) 15 mg/kg IV on day 1 of every 90-day cycle.	Metastatic CRC, pretreated with any or combination of 5-FU, oxaliplatin, irinotecan and cetuximab (*n* = 47 patients)	PR : 1 patient PD: 43 patients	4.8 months (95% CI, 4.1 to 7.7 months)	PFS : 2.3 months (95% CI, 2.1–2.6 months)	No clinically meaningful single-agent activity

CR = complete response; CRC = colorectal cancer; CTLA-4 = cytotoxic T-lymphocyte-associated protein 4; IV = intravenous; L1 = programmed death ligand 1; OS = overall survival; PD = progressive disease; PD-1 = programmed death 1; PD-MMR = mismatch repair; PFS = progression-free survival; PR = partial response; RR = response rate; SD = stable disease

### Anti-PD-1/PD-L1

The anti–PD-1 antibody nivolumab did not demonstrate clinically significant activity in a Phase I study of multiple tumor types, which included 17 patients with heavily pre-treated, metastatic CRC. However, six of the seven tumors found in this cohort, which were tested for PD-L1 expression, were negative, potentially explaining the observed lack of response [37]. Notably, one patient from this metastatic-CRC cohort, who had a PD-L1 positive tumor and was treated with 5 doses of nivolumab, showed complete response after 6 months and no signs of disease after 3 years. This patient’s tumor was also mismatch-repair-deficient [42].

Pembrolizumab is a humanized IgG4 monoclonal antibody that blocks the interaction of PD-1 with its ligands PD-L1 and PD-L2. A Phase II trial of pembrolizumab evaluated 32 patients with advanced CRC [11 with deficient mismatch repair (dMMR) and 21 with proficient mismatch repair (pMMR)] [43]. All patients enrolled in the study had received two or more (median of four) previous chemotherapy regimens, except one patient who had received one chemotherapy regimen and one non-PD-1 based immunotherapy before. The immune-related objective response rate and patients with stable disease were significantly higher in the dMMR CRC patients (40% and 78%, respectively) than in the pMMR CRC patients (0% and 11%, respectively). Similar findings were observed in the cohort with dMMR cancers other than CRC. Based on this a larger, Phase II study (Keynote-164) is currently enrolling patients to assess the benefit of pembrolizumab for unresectable or metastatic dMMR CRC refractory to two or more previous lines of therapy (NCT02460198).

The anti–PD-L1 antibody BMS936559 showed no activity in a Phase I trial that included 18 patients with CRC [44]. However, preliminary data with another anti–PD-L1 antibody MPDL3280A showed activity in CRC with one of four patients achieving a durable partial response [45]. Another Phase Ib trial, assessing MPDL3280A in combination with bevacizumab in refractory CRC patients and in combination with FOLFOX plus bevacizumab in first-line CRC, showed good tolerability and clinical activity [unconfirmed ORR 8% (1/13) and 44% (8/18), respectively] [46].

### Mismatch repair deficiency and checkpoint inhibition

A dMMR system is present in 10–20% of patients with sporadic CRC and is associated with a favorable prognosis in early-stage disease. In contrast, dMMR occurs in only 3–6% of patients with advanced CRC [47, 48]. As the immune system can recognize somatic mutations found in tumors [49]—and as colorectal tumors with dMMR have several times as many somatic mutations as proficient MMR tumors [50, 51]—it has been hypothesized that the immune system may play a role in dMMR tumors possessing a reduced metastatic potential. Additionally, dMMR cancers have prominent lymphocyte infiltrates [52, 53], which supports the above hypothesis. Furthermore, recent findings have suggested that the infiltrate in dMMR CRC is more likely to express PD-L1, which may predict response to PD-1 blockade [54]. Thus checkpoint inhibitors may have increased activity in dMMR tumors, a hypothesis which was tested in a Phase II trial. This study showed that MMR status may be a predictive biomarker for clinical benefit from checkpoint inhibition [43]. Although immunotherapy in dMMR tumors holds great promise, the complete absence of response in patients with pMMR CRC—who represent the vast majority—highlights the ongoing need to understand why patients with conventional CRC lack robust responses to immunotherapy.

### CTLA-4

Tremelimumab is a fully human IgG2 monoclonal antibody that blocks the inhibitory action of CTLA-4, and was tested as a monotherapy in a single-arm, Phase II trial in 47 patients with refractory metastatic CRC [55]. All the patients were heavily pre-treated and most had also received cetuximab. Tremelimumab did not show significant single-agent activity in this study, which could be due to delayed immune responses in the setting of advanced CRC, since 43 of 45 patients experienced disease progression with a median duration on study of only 2.3 months. The first FDA-approved CTLA-4 monoclonal antibody, ipilimumab, is currently being evaluated in combination with nivolumab (anti-PD-1 antibody) in clinical trials for metastatic CRC patients with dMMR tumors (NCT02060188).

### Other immune checkpoint inhibitor*s*

Tumors with mismatch repair deficiency also have a high expression of immune checkpoint ligands such as PD-L1, PD-1, CTLA-4, and LAG-3, which effectively counterbalance the active Th1 response and prevent tumor elimination. These immune checkpoints function as possible targets for inhibition during cancer immunotherapy [56]. Anti-LAG-3 monoclonal antibodies (BMS-986016), alone and in combination with nivolumab, are already undergoing evaluation in clinical trials (NCT01968109). These are promising future targets for immune checkpoint inhibition.

## Vaccine-based immunotherapy

### Whole-tumor-cell vaccines

The earliest attempts to generate anti-CRC immune responses were in the form of whole-tumor-cell vaccines. Most studies have used autologous CRC tumor cells in combination with Bacillus Calmette-Guérin (BCG). The Eastern Cooperative Oncology Group (ECOG) E5283 was a randomized, Phase II I trial that randomly assigned 412 patients with resected stage II and III colon cancer to an observation arm or a treatment arm with autologous tumor-cell-BCG vaccine. After a median follow-up of 7.6 years, no statistically significant differences were seen in terms of clinical outcomes but effective immune responses—observed as delayed cutaneous hypersensitivity (measured as skin induration)—correlated positively with disease-free survival (DFS) and OS [57]. Another trial assessed the efficacy of three post-operative vaccinations, followed by a booster dose 6 months later, in 254 resected stage II and III CRC patients. The study showed a 44% reduction in risk of recurrence in the vaccine group [58]. This study showed the importance of vaccine quality control for proper immunogenicity, as 12% of vaccines in the ECOG trial failed to meet quality control specifications. Delayed cutaneous hypersensitivity was also shown to be an effective surrogate endpoint in monitoring the long-term efficacy of tumor cell vaccines [59]. A meta-analysis of all trials evaluating this strategy in resected stage II and stage III CRC reported a significant improvement in OS (HR = 0.76; *P* = 0.007) and DFS (HR = 0.76; *P* = 0.03) [59].

As observed in early trials, autologous whole-tumor-cell vaccines were poorly immunogenic, which was probably due to paucity of tumor-associated antigens (TAAs) in whole-cell vaccines and the large proportion of antigens shared with normal cells [60]. Another tumor-cell vaccine approach, utilizing Newcastle disease virus-infected, irradiated, autologous tumor cells (ATV-NDV) was tested in a Phase II trial. Of patients treated with ATV-NDV, 61% developed tumor recurrence, compared with 87% treated with surgery alone [61]; however, in terms of overall survival, a Phase II I trial with 50 patients failed to show significant improvement for ATV-NDV-treated patients, although a sub-group analysis showed benefit for colon—but not rectal—cancer patients [62].

GVAX, a GM-CSF gene-transfected tumor-cell vaccine, has shown immune-stimulatory action in several cancers [63]. There is a current pilot study using GVAX in irradiated autologous CRC cells in patients with stage IV CRC, which examines safety as a primary endpoint, as well as progression-free survival (PFS) and OS (NCT01952730).

### Peptide vaccines

Peptide vaccines incorporate one or more short or long amino acid sequences as tumor antigens, combined with a vaccine adjuvant. Several TAAs expressed by colorectal cancer cells—such as carcino-embryonic antigen (CEA), MUC-1, survivin, beta-human chorionic gonadotropin (hCG) and ring finger protein 43 (RNF43)—have been targeted via peptide vaccines. Results have been modest, with only a few patients showing clinical response, despite demonstration of antigen-specific T-cell responses following vaccination.

A Phase I safety study, using plasmid DNA vaccine-encoding CEA (CEA66 DNA) alongside GM-CSF in CRC patients, showed good tolerability with no signs of autoimmunity [64]. In another Phase I trial, HLA-A24-specific survivin-2B vaccine was administered to 15 patients with advanced or recurrent CRC expressing survivin, with one patient showing an increase in survivin-specific CTLs and six showing decreased CEA levels [65]. A cohort of 21 CRC patients, treated with a peptide-cocktail vaccine derived from RNF43 and translocase of the outer mitochondrial membrane 34 (TOMM34), showed antigen-specific CTL responses to both RNF43 and TOMM34 in 8/21 and CTL response to only one of the peptides in 12 out of 21 patients. Interestingly, survival rates were lowest in the patients who showed no CTL responses [66]. Another TAA of interest is hCG, which is not produced by normal colorectal epithelial and mucosal cells or benign lesions. Moulton *et al*.** studied the effect of hCG peptide vaccination in 77 patients with stage IV CRC, most of whom had a history of chemotherapy with one or more agents; anti-hCG antibodies were observed in 56 out of 77 patients and a longer survival was seen in patients with higher-than-median levels of antibody [67].

### Viral vector vaccines

The immunogenicity of peptide vaccines is low, and could be amplified by packaging the peptide in a viral or bacterial vector. Several groups have attempted to use immunogenic viral vectors expressing CRC TAAs to stimulate anti-CRC immune responses. A Phase I study with ALVAC-CEA, a canary pox virus expressing CEA and B7.1 co-stimulation, exhibited an acceptable safety profile in 118 patients. Objective responses were seen in 42 (40%) patients, whereas another 42 (40%) had stable disease, with several patients demonstrating CEA-specific T-cell responses [68]. Similar results were seen with a recombinant fowl pox virus expressing multiple co-stimulatory molecules (B7.1, LFA-3, ICAM-1) and the CEA antigen—administered alone or in sequence with a recombinant vaccinia virus expressing the same molecules—to patients with a variety of CEA-expressing tumors, including 35 patients with CRC. Most patients showed an increase in CEA-specific T-cells, and durable disease stability of at least 4 months was observed in 40% of patients (23 out of 58).

### Dendritic cell vaccines

Dendritic cells (DCs) are crucial for cell surface presentation of endogenous and exogenous immunogenic peptides and activation of T-cells. Various strategies for delivering TAAs to DCs through synthetic peptides [69], tumor RNA [70], and tumor cell lysates [71] have been developed to stimulate an adequate CTL response. Phase I clinical studies using autologous human DCs, pulsed with CEA peptide, in CEA-expressing metastatic CRC patients showed the vaccine to be safe and well tolerated, although only a modest clinical improvement was observed [72]. Another Phase I study used CEA-derived peptide loaded onto DCs, plus a vaccine adjuvant (Flt3-ligand). After vaccination, 2 of 12 patients experienced dramatic tumor regression, one patient had a mixed response, and two exhibited stable disease [73]. Antigen selection remains a key issue with these dendritic cell–based vaccination approaches, and overall success rates have been modest.

## Adoptive cell transfer therapy

Adoptive cell transfer therapy (ACT) is a technique in which autologous T-cells are extracted from patients, activated, expanded *in vitro* and re-injected into patients. Adoptive cell therapies have shown some activity in CRC, using autologous T-cells genetically engineered to express high-affinity receptors for CRC TAAs. ACT enables the selection and activation of highly reactive T-cells, with clinical responses observed even in advanced CRC [74]. In a Phase I study, autologous T-cells, genetically engineered to express a high-affinity murine T-cell receptor against human CEA, were administered to three patients with refractory CRC. Serum CEA levels decreased significantly (74–99% reduction) in all patients and one patient exhibited an objective decrease in tumor size. But further enrollment of patients was halted because all three patients developed autoimmune colitis (two patients with grade 3) [75]. This was the first study in which an objective regression was noted in metastatic colorectal cancer through ACT. Morgan *et al*.** used HER2-specific chimeric antigen receptor (CAR) T-cells for patients with metastatic CRC, but failed to demonstrate its safety and efficacy due to severe side-effects [76]. Further studies on this front could possibly lead to safe and efficacious ACT therapy for CRC. Various trials are in progress in this field of investigation, including a Phase I dose escalation of infusion of CTLs specific for TAAs NY-ESO-1, MA-GEA4, PRAME, Survivin, and SSX in relapsed or refractory solid tumors (NCT02239861), and a Phase I/II study of the safety and efficacy of infusion of peripheral blood lymphocytes transduced with a CAR that is specific for VEGF-2 in metastatic CRC following a lympho-depleting conditioning regimen with cyclophosphamide and fludarabine (NCT01218867).

## Special considerations with immunotherapy

The kinetics of clinical responses with immunotherapy can be very dissimilar to conventional therapies such as chemotherapy. A shared feature of several of the approved immune therapies (in particular cytokines and checkpoint inhibitors) is that only a small fraction of patients have early objective response using standard RECIST criteria. It is not uncommon for some lesions to grow prior to regression, and patients may often demonstrate a lag in apparent clinical response. Specific immune-related response criteria have been developed to overcome this shortcoming. These criteria allow for some interval progression, as long as the patient is not deteriorating significantly [77]. These criteria should be routinely incorporated into immunotherapy clinical trials for CRC.

Another crucial question is the most appropriate clinical setting in which to study immunotherapy in CRC. Although the time lag in clinical response may suggest using these earlier in the course of metastatic CRC—or possibly even in neoadjuvant or adjuvant setting—currently approved indications for immunotherapies in melanoma and non-small cell lung cancer are for advanced disease often pre-treated with chemotherapy. Most of the current investigations are being carried out in advanced CRC; a few trials combining checkpoint inhibition with up-front FOLFOX chemotherapy are being evaluated for first-line treatment of metastatic CRC (NCT02375672).

Immunotherapies are also associated with some peculiar auto-immune adverse events, such as colitis, hepatitis, and endocrinopathies for checkpoint inhibitors such as ipilimumab and nivolumab [8, 37]. These toxicities appear to be less pronounced with the PD-1/PD-L1 inhibitors, although pneumonitis has been reported with these agents. Toxicities with PD-1/PD-L1 are generally manageable with corticosteroids and tumor necrosis factor (TNF)-α inhibitors.

## Future directions

The approval of more drugs in this novel class of immune checkpoint inhibitors—as well as development of several newer immunotherapies that are undergoing testing in clinical trials—would continue to provide innovative insights relevant to future investigations of CRC immunotherapy. Although PD-L1 expression and dMMR tumors are currently showing promise as predictive biomarkers for response to checkpoint inhibitors, it is necessary to identify specific patient sub-groups, in the appropriate clinical setting, that would benefit most from immunotherapy.

It is also imperative to study rational combinations involving immunotherapy drugs. Given the evidence for immunogenic cell death triggered by oxaliplatin, the combination of first-line FOLFOX chemotherapy and checkpoint inhibition holds significant promise. As checkpoint inhibitors are thought to enhance pre-existing immune response to tumor antigens by releasing tumor-induced inhibition of the immune response, combinations of antigen-directed therapies (e.g. vaccines), sequentially followed by checkpoint inhibition, may also be a rational approach in CRC.

Another novel strategy being currently evaluated involves stimulation of anti-tumor immune response using urelumab that activates co-stimulatory CD137 receptor. Combining this approach with checkpoint inhibition (activate co-stimulatory receptors and inhibit co-inhibitory receptors) appears promising and a Phase I trial of urelumab in combination with nivolumab is currently enrolling subjects (NCT02534506). Another clinical trial of urelumab in combination with cetuximab is also underway in metastatic CRC patients (NCT02110082).

## Conclusion

Current clinical trials of immunotherapy in CRC—specifically checkpoint inhibitors—are already suggesting its efficacy in carefully selected subsets of patients with CRC. Developing reliable, predictive biomarkers and understanding the mechanisms that underlie lack of response or resistance to these agents remains a challenge. Based on their demonstrated effectiveness in a broad range of solid malignancies, immunotherapies hold great promise in CRC.

*Conflict of interest statement*: none declared.
